# 11th IUBMB Focused Meeting on the Aminoacyl-tRNA Synthetases: Sailing a New Sea of Complex Functions in Human Biology and Disease

**DOI:** 10.3390/biom8020022

**Published:** 2018-05-01

**Authors:** Christopher Francklyn, Herve Roy, Rebecca Alexander

**Affiliations:** 1 Department of Biochemistry, College of Medicine, University of Vermont, Burlington, VT 05401, USA; 2 Burnett School of Biomedical Sciences, College of Medicine, University of Central Florida, Orlando, FL 32827, USA; herve.roy@ucf.edu; 3 Department of Chemistry, Wake Forest University, 1834 Wake Forest Road, Winston-Salem, NC 27109, USA

**Keywords:** aminoacyl-tRNA synthetases, genetic code, translation, protein synthesis

## Abstract

The 11th IUBMB Focused Meeting on Aminoacyl-tRNA Synthetases was held in Clearwater Beach, Florida from 29 October–2 November 2017, with the aim of presenting the latest research on these enzymes and promoting interchange among aminoacyl-tRNA synthetase (ARS) researchers. Topics covered in the meeting included many areas of investigation, including ARS evolution, mechanism, editing functions, biology in prokaryotic and eukaryotic cells and their organelles, their roles in human diseases, and their application to problems in emerging areas of synthetic biology. In this report, we provide a summary of the major themes of the meeting, citing contributions from the oral presentations in the meeting.

## 1. Introduction

The aminoacyl-tRNA synthetases (ARSs) catalyze the first reaction of protein synthesis, namely the attachment of amino acids to the 3′ ends of cognate tRNAs. The fundamental chemistry of the aminoacylation reaction and the existence of specific enzymes to catalyze it have been known about for more than 50 years. It is of note that early pioneers of biochemistry and molecular biology were quick to appreciate that ARSs have a particularly difficult task as enzymes, and this helped to define some of the initial questions that propelled much of the early work on these enzymes [1,2,3,4]. Essentially, a full understanding of how the genetic code is implemented at the molecular level demanded that we establish how ARSs efficiently select the correct amino acids and tRNAs for aminoacylation, and then join them at rates sufficient for protein synthesis. Owing to the challenging discrimination problem this entails, it is not surprising that ARSs were among the first enzymes for whom bona fide editing/proofreading reactions could be demonstrated [3,4]. While resolving these early questions would frame much of the research effort since the 1960s, a recurring theme of the tRNA synthetase field is that, just when the scientific community is ready to relegate these ancient enzymes to the back chapters of biochemistry textbooks, they provide new surprises that overturn the established scientific canon.

For many enzyme superfamilies, the evolution of diverse forms from a single ancestral catalytic scaffold provides a satisfactory solution to the requirement for functional variability, including the ability to recognize many different structural variations of a basic substrate. In 1990, a pivotal year in ARS research, several labs established that there are in fact two distinct ARS classes, each with a distinct catalytic fold, characteristic peptide sequence motifs, and regiochemistry of aminoacylation [5,6]. This discovery heralded the first specific conference on the aminoacyl-tRNA synthetases in Autrans, France, beginning a tradition of periodic gatherings in various locations in Europe, Asia, and the United States. The 11th such conference was recently held in Clearwater Beach, Florida, in late 2017. In areas such as the structure, evolution, and catalytic function of these enzymes, a clear line can be drawn from the Autrans meeting to the Clearwater meeting. However, many of the developments discussed in Clearwater could scarcely have been predicted at the first ARS meeting in Autrans.

One of the principal takeaways from the Clearwater meeting is that that the field has evolved from an early focus on the structural and mechanistic details of amino acid-ARS-tRNA complexes to embrace the knotty issue of how the enzymes function in the broader context of the cell and the organism as a whole. Here, we will review the Clearwater meeting with a focus on seven major themes that serve to organize the presentations of the meeting and highlight how these underscore key future directions in the field. These themes consist of: (1) complexity; (2) mechanism/fidelity; (3) spatial/temporal diversity; (4) signaling functions; (5) disease connections; (6) therapeutics; and (7) synthetic biology. While it is difficult to extract one simple overriding message from the meeting, the emerging picture is that, in addition to providing key precursors to protein synthesis, many of the ARSs act to communicate information about the environmental and nutritional status of the cell to the transcriptional, translational, and other complex machineries resident in various compartments. Such interactions may help to maintain proteostasis and homeostasis.

## 2. Complexity

One of the key insights that has been gained from comparative studies of the human and mouse genomes is that both encode a relatively small number of open reading frames, estimated at 20,687 in the case of the human. However, the complexity of the proteome is far greater than the number of encoded open reading frames for several reasons [7]. First, proteins can be altered at diverse positions with one or more of a growing set of potential post-translational modifications (including phosphorylation, acetylation, ubiquitination, and others) and these clearly modify catalytic activity, cellular localization, and protein lifetime. Second, the approximately 20,000 different human protein coding genes can be differentially spliced during expression to generate more than 100,000 different isoforms, many of which are restricted to specific cell type or whose expression is limited to specific times during development. Last, there is sufficient variability in the genome among individuals to both alter the levels of expressed proteins and create the possibility of novel interactions.

In his opening keynote lecture, Paul Schimmel (The Scripps Research Institute) emphasized how the tRNA synthetases epitomize many of the aforementioned complexity features of the human proteome. In earlier work, his group analyzed how the gradual accretion of accessory domains by tRNA synthetases as they move up the evolutionary ladder allowed them to take on an increasingly complex portfolio of functions distinct from translation [8]. More recently, his lab showed that many ARS genes undergo alternative splicing to generate versions lacking the catalytic domain; some of these display activity in biological assays for proliferative, immunomodulatory, and inflammatory functions [9]. In his presentation, Schimmel noted how accumulated mass spectrometry has revealed that each human ARS interacts with more 15 other intracellular partners, providing evidence of an extensive ARS interactome, likely devoted to more than translation. Other work focused on studies of inherited compound heterozygous mutations in the human mitochondrial phenylalanyl-tRNA synthetase (*FARSB*). A key take home message from the talk is that rather than representing a simple loss-of-function effect, the pathogenic mutation leads to wide scale changes in the overall cellular transcriptome.

The theme of complexity in the ARS proteome was carried on in other presentations, and these exemplified how changes in the post translational modifications affect ARS function and cellular localization. Earlier studies had shown that under oxidative stress, human tyrosyl-tRNA synthetase (*YARS*) translocates to the nucleus where it activates the transcription factor E2F to increase transcription of DNA repair genes [10]. New work presented showed that, in response to oxidative stress, *YARS* becomes acetylated at K244, promoting nuclear translocation and subsequent E2F interaction (Wei Yu, Fudan University). The extent of modification is determined by the balance of activities between p300/PCAF, which possesses acetylation function, and SIRT1, which removes the acetylation mark. The tyrosyl-tRNA synthetase in *Escherichia coli* is similarly modified by acetylation at lysines 85, 235, and 238, likely by the action of members of the GCN5-related family of *N*-acetyl transferases. The use of genetic code expansion strategies allowed the functional consequences of acetylation to be tested, revealing that acetylation decreases aminoacylation (Chenguang Fan, University of Arkansas) [11]. Another example of a functionally important post-translational modification (PTM) is the nitric oxide induced *S*-nitrosylation of human mitochondrial threonyl-tRNA synthetase (mTARS), which inhibits both aminoacylation and editing activities (Xiao-Long Zhou, Shanghai Institute of Biochemistry and Cell Biology). Interestingly, mTARS appeared not be modified by hydrogen peroxide, which has been shown to impair the editing activity of *E. coli* threonyl-tRNA synthetase and promote mistranslation [12,13]. Other examples of complexity in the tRNA synthetase proteome illustrated how loss and gain of specific domains alter the canonical function. In other studies on *YARS*, differences in alternative splice forms were correlated with different tissue types. One special splice form is produced during adipogenesis of mesenchymal stem cells and could be co-immunoprecipitated with the nuclear poly (ADP-ribose) polymerase (PARP) (Zhiwen Xu, HKUST). With regard to gain of new domains, a plant version of histidyl-RNA synthetase was described that contains an appended domain homologous to histidine ammonia lyase that contributes to anthocyanin and lignin biosynthesis (Tetsuo Kushiro, Meiji University). These are likely to represent only a few examples of how the ARS proteome can be reshaped to achieve functional flexibility under varying environmental conditions.

## 3. Mechanism/Fidelity

One of the earliest and most intensively studied problems in the ARS field is how discrimination between chemically similar amino acids is achieved. For the isolated enzymes in solution, key aspects of the problem were resolved by discoveries of specialized editing domains in a number of the ARS families (IleRS, ValRS, LeuRS, and ThrRS) [14,15,16,17], and by the elucidation of the kinetic proofreading mechanisms that dictate how the various editing pathways are utilized [18,19]. These successes lead us to pose a more difficult question: how important are ARS editing mechanisms to the maintenance of normal cellular physiology, and to the pathophysiology of human diseases? A vivid example of the neuroprotective effect of ARS editing function was described by Susan Ackerman (UC San Diego) in her presentation on how Ankryin Repeat Domain 16 (ANKRD16) acts to suppress the neurodegenerative phenotype associated with an alanyl-tRNA synthetase (AARS) mutation (the AARS linked “sticky” allele [20]) in an inbred mouse strain that attenuates editing function. Through a careful set of genetic and biochemical experiments, Ackerman and her collaborators showed that ANKRD16 is post-translationally modified by addition of misactivated serine produced by the mutant AARS, thereby decreasing the amount of serine misincorporation (and presumably, the burden of misfolded proteins) in the Purkinje cells that are targeted by the sticky allele. While the ANKRD16 suppressor appears to specifically rescue serine misincorpration, how is potential miscoding by glycine addressed? This question has now been elegantly resolved by the discovery of catalytic promiscuity on the part of d-aminoacyl-tRNA deacylase. Owing to the lack of chirality in glycine, it too can be efficiently removed from tRNA^Ala^ by the d-aminoacyl-tRNA deacylase when mis-acylated by mutant AARS (Rajan Sankarayananan, CCMB Bangalore) [21].

In the sticky mouse, therefore, loss of editing function clearly results in a decrease in genetic fitness. In other cellular contexts, responses to the generation of misacylated tRNA can have a variety of effects. For example, the *E. coli* PheRS has difficulty accommodating m-(oxidized)-Tyr in its editing domain, and the lack of editing can decrease cellular viability. Notably, reduced tyrosine editing by yeast PheRS appears to attenuate the ability of yeast to mount a robust unfolded protein stress response (Mike Ibba, Ohio State). By contrast, mis-methionylation of non-cognate tRNAs may help eukaryotic cells mitigate high levels of oxidative stress [22], as does the oxidative deactivation of ThrRS editing in *E. coli*. In arguably the most dramatic example of the benefit of mistranslation, elevated mis-serylation of non-cognate tRNAs has been shown to enhance the pathogenicity of *Saccharomyces cerevisiae* and *Candida albicans* [23]. These observations highlight the need for improved tools to study mistranslation, including improved mass spectrometry approaches and assays for measuring quality control processes in single cells (Jiquing Ling, U. Texas).

## 4. Spatial/Temporal Diversity

During the initial decades of tRNA synthetase research, the rapid growth and simplicity of *E. coli* made it the preferred model organism for many studies. With limited exceptions, bacterial ARSs reside in a single compartment, and organelle-specific enzymes do not exist in prokaryotes. In eukaryotes, there are distinct cytoplasmic and mitochondrial tRNA synthetases for most ARS families (for the *KARS* and *GARS* families a single gene provides both versions); essentially all ARS genes are encoded in the nuclear genome. Consequently, diverse mechanisms, such as alternative splicing and alternative translational start sites, are required to ensure that organellar targeting sequences are appended to the tRNA synthetases to commit them to the mitochondria or other, more specialized organelles like chloroplasts. In higher eukaryotes, 11/20 of the cytoplasmic ARSs and three accessory proteins are organized into a multisynthetase complex (MSC) whose organization may promote channeling of aminoacylated-tRNAs to ribosomes and yet also facilitate tighter integration of signaling pathways to control of protein synthesis (see Signaling, Section 5). Notably, the cytoplasm, mitochondria, and chloroplast all represent translationally active compartments. To add further complexity, there is also evidence that a number of tRNA synthetases localize to the nucleus under specific physiological conditions. Nuclear resident ARSs may be involved in a tRNA maturation quality control process, or other functions distinct from translation. Defining how migration of ARSs among cellular compartments influences protein synthesis and other ARS functions requires new cell biology tools. One potentially valuable approach shared at the meeting involves the attachment of split green fluorescent protein GFP tags to ARSs, which produce a fluorescent signal when linked to the complementary GFP fragment located in the target compartment (Hubert Becker, U. Strasbourg). By this method, researchers will now be able to track “relocating” ARSs that move in and out of various cellular compartments, even when only a fraction of the available ARS undergoes migration.

Owing to their strong connection to human diseases (see Disease, Section 6), mitochondrial ARSs continue to attract strong research interest. Mitochondrial ARS-tRNA interactions often feature substantial differences in the nature of specificity-determining identity elements relative to their cytoplasmic counterparts (Joe Chihade, Carleton College). As more and more clinical reports accumulate, a major effort in this area is to generate a comprehensive database that correlates all known mitochondrial ARS and mitochondrial tRNA mutations with the affected organ systems (Marie Sissler, CNRS Strasbourg) [24]. While many of the mutations identified in mitochondrial ARSs clearly lead to a compromise of aminoacylation function, interesting exceptions have been noted. In the mitochondrial arginyl-tRNA synthetase (mRARS), there is evidence that part of the wild type function involves localization to the inner membrane of the mitochondrial matrix, and that disease-linked mutations affect mitochondrial development. Membrane association by ARSs is not limited to mitochondria. In the cyanobacteria *Anabeana sp* PC7120, a CAAD domain appended to the C-terminus of ValRS confers anchorage to the thylakoid membranes of the chloroplast (Ignacio Luque, University of Seville). Intriguingly, in this location ValRS interacts with elements of the F1 ATPase; the functional consequences of this interaction remain to be identified. One model to explain the ValRS-F1 ATPase interaction is that the synthetase may be serving as a valine sensor, providing information on nutritional status. Other presentations highlighted the potential for ARSs to exhibit sensing functions. In *E. coli* under nutritional stresses, a shortened form of LeuRS can be detected in the periplasmic space, and a fraction of this (along with tRNA) appears to be secreted into the media (Susan Martinis, University of Illinois at Urbana-Champaign). Fly species (*Drosophila* and mosquitoes) contain an unusual SerRS paralog (SLIMP) that appears to coordinate the cell cycle with mitochondrial anabolism [25]. SLIMP can stimulate aminoacylation by SerRS, but also promotes degradation of the transcription factor gene-derived microsatellite (TFQM) transcription factor by the Lon protease (Lluís Ribas, Institute for Research in Biomedicine Barcelona). Consequently, replication of mitochondria is inhibited, and cell cycle progression halted by activation of G2/M checkpoint controls. In the mosquito, down-regulation of SLIMP increased the number of mitochondria, reduced respiratory rates, and increased the generation of reactive oxygen species (Gilbert Oliveira, Universidade Federal Rio de Janeiro).

## 5. ARS as Mediators of Cellular Signaling

ARSs have been known for over a decade to be important as signaling agents, and these activities can involve a number of the cellular compartments discussed above. A key set of recently established functions for ARSs involve metabolic sensing. The mammalian target of rapamycin (mTORC) is a global cellular regulator that responds to changes in amino acid levels, particularly leucine. High levels of leucine promote mTORC translocation to the lysosome, bringing it in contact with the RagABCD GTPases and the RAPTOR scaffolding protein. Recent studies have suggested that human leucyl-tRNA synthetase (LARS) serves as a leucine dependent GTPase activating protein for the Rag GTPases [26], but other groups have proposed models involving the Sestrins [27]. Work presented at the meeting described small molecule inhibitors of this LARS function, and also described a new, more comprehensive model in which LARS and the Sestrins both participate in leucine sensing (Jun Min Han, Yonsei University). In addition to LARS, other tRNA synthetases that may be involved in anabolic signaling include glutamyl-prolyl tRNA synthetase (EPRS) and TARS. A key target of activated mTORC1 is the S6 kinase, and new work suggests that under the influence of insulin signaling, mTORC1-S6 phosphorylates the WHEP linker domain of EPRS leading to its release from the multisynthetase complex and stimulation of adipogenesis (Paul Fox, Cleveland Clinic). The liberated EPRS then binds to fatty acid transporter SLC27A1, inducing its translocation to the plasma membrane and long-chain fatty acid uptake. By contrast, other new data was presented arguing that human threonyl-tRNA synthetase (TARS) can interact with the EIF4E2 component of the eukaryotic translation initiation complex to promote translation under hypoxic conditions (Sung Kim, Seoul National University). Intriguingly, these results may account in part for the apparent ability of TARS to promote angiogenesis, which is known to be upregulated during hypoxia [28]. Finally, a remarkable new finding is that mitochondrial cysteinyl-tRNA synthetase (CARS) catalyzes the production of cysteine hydropersulfide (CysSSH), both on free cysteine and on the cysteines of a broad range of target proteins, including CARS itself (Taka Akaike, Tohoku University). Unexpectedly, CysSSH can function as a terminal electron acceptor in mitochondrial respiration, suggesting that this function is deeply imbedded in global cellular redox control.

In additional to sensing the nutritional and metabolic state, ARS signaling functions are of growing importance in the nucleus and in response to viral infection. As noted above, tyrosyl-tRNA synthetase relocates to the nucleus in response to oxidative stress, and this relocation is driven by acetylation. There appear to be a number of interesting functional consequences of nuclear relocalization (a third is described under Diseases). One major consequence is that it promotes down-regulation of protein synthesis, and the mechanism appears to involve recruitment of chromatin modifying enzymes to the promoters of important translation factors, including ARS genes and elongation factors like EEF1A1 (Na Wei, The Scripps Research Institute). A further dimension of the YARS story is that, in response to binding resveratrol, its nuclear functions also include activation of PARP, which has important global roles in repair of DNA damage (Sajish Mathew, University of South Carolina). A potentially significant consequence of this interaction is that it may inhibit Sirtuin 1 and thereby regulate global protein acetylation. Another well-characterized example of nuclear tRNA synthetase function is exemplified by lysyl-tRNA synthetase (KARS). Previously, phosphorylation of KARS at Ser207 was shown to promote release from the multisynthetase complex and nuclear translocation. In the nucleus, KARS binding to Microphthalmia-associated transcription factor (MITF) stimulates Ap4A production and the activation of transcription by the transcription factor MITF [29]. An important new development places the nuclear translocation of KARS downstream of activation of the Epidermal Growth Factor Receptor (EGFR). Of note, small cell lung cancer patients with mutant versions of EGFR exhibited a greater extent of nuclear phospho-S207 KARS staining than patients with non-mutant receptor, and improved disease-free survival (Hovav Nechushtan, Hadassah Hebrew University). The nuclear translocation of pSer207 KARS also promotes the infectivity of HIV-1 via mechanisms that are not yet clear; interestingly, translocation does not seem to control the packaging of KARS into HIV virions, where it contributes to HIV replication (Karin Musier-Forsyth, Ohio State). In contrast to KARS, whose secondary functions promote viral replication, EPRS is part of the antiviral response (Myung Hee Kim, Korea Research Institute of Bioscience & Biotechnology) [30]. Upon infection, EPRS is phosphorylated at Ser900, released from the multisynthetase complex, and binds to poly C binding protein 2 (PCBP2). This serves to prevent PCBP2 from mediating the degradation of the mitochondrial antiviral-signaling (MAVS) proteins, a critical positive mediator that activates innate immune response to viral RNA. The up-regulation of EPRS upon viral infection is counter to the responses of other ARSs, whose expression is inhibited. These examples underscore the diverse pathways in which ARSs contribute to cellular responses to oxidative stress, changes in nutritional state, and viral infection. Despite all that has been recently learned, the unanswered question remains: why were the tRNA synthetases chosen for these functions?

## 6. tRNA Synthetases and Human Diseases

**Figure 1:**
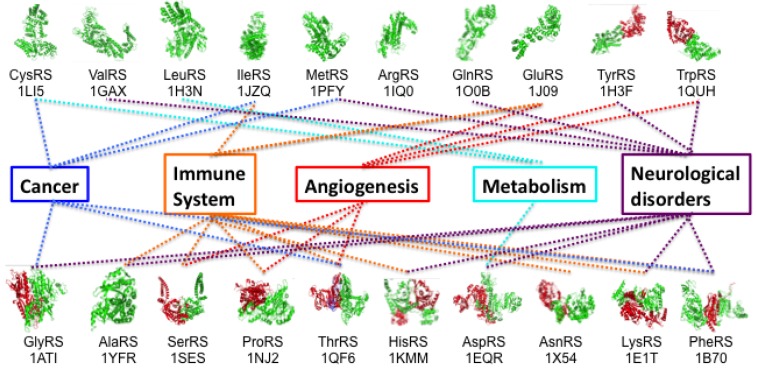
Links between aminoacyl-tRNA synthetases, human physiology and disease suggested by current research. Further details about these specific associations can be found in recent reviews from investigators in the tRNA synthetase field [24,32,33,34].

A major consequence of the widespread availability of rapid whole-exome sequence technologies has been that the time and effort required to identify genes linked to human diseases have been drastically reduced. The number of disease-causing alleles in human tRNA synthetases has risen dramatically, and for all but four of the 37 ARS genes there is at least one corresponding disease [31]. Accompanying this wealth of new information (Figure 1) is a major research question that many labs are now grappling with: given that all tRNA synthetase families (and their respective cytoplasmic and mitochondria enzymes) are required for protein synthesis in all cells and tissues, what accounts for the specialized (often linked to the peripheral or central nervous system) nature of ARS associated diseases? As part of the strategy to address this question, strong collaborations are developing between basic scientists and clinicians, with both partners educating each other.

The Clearwater meeting marked one of the first occasions where ARS scientists had the opportunity to hear directly from clinicians treating patients with tRNA synthetase related diseases. Charcot Marie Tooth (CMT) disease is a chronic progressive neurodegenerative phenotype that leads to muscle wasting and a loss of motor neuron function, affecting the ambulatory and proprioception functions of patients. Charcot Marie Tooth is of particular interest to the ARS community owing to the fact that mutations in the genes encoding glycyl-tRNA synthetase (GARS), YARS, AARS, histidyl-tRNA synthetase (HARS), and tryptophanyl-tRNA synthetase (WARS) have all been linked to the disease, exclusively as a dominant condition. In his keynote presentation, Michael Shy (University of Iowa) described the work of his CMT peripheral neuropathy clinic, illustrating with short movies how the simple task of walking is challenging for these patients. For scientists wishing to understand how specific ARS mutations lead to disease presentations of varying severity, a systematic way to classify and score patients is essential. This is a major effort of the larger CMT consortium in which Dr. Shy is a major contributor; other classification efforts feature the use of magnetic resonance imaging (MRI) to detect the conversion of myocytes to adipocytes that occurs in most patients. To complement the valuable CMT clinical work, ARS researchers are employing classical biochemical approaches to study mutant proteins, and also yeast, *C. elegans*, and mouse models to evaluate the mutations for pathogenicity and to define the molecular mechanisms of disease. An interesting result of these analyses is a spectrum of functional consequences that gives rise to a range of clinical phenotypes ranging from mild neuropathy to severe, lethal, recessive, developmental syndromes (Anthony Antonellis, U. Michigan). Analysis of an allele of YARS that appears not have lost aminoacylation in a Drosophila model suggests that the mutation might be affecting nuclear specific functions of YARS, including an effect on E2F transcription (Sven Bervoet, University of Antwerp). Another model system that holds great promise for investigating ARS neurological disease is the zebrafish. One experimental approach is to depress the expression of the wild-type gene by injecting the embryos with a splice-directed morpholino, and then complement by co-injecting with mRNA encoding an ARS mutation (Marian Wederman, Leiden University Medical Center). Preliminary analysis suggests that developing zebrafish carrying the mutant alleles exhibit defects in neural development. Mouse models are critical for the study of ARS-linked CMT, as they can provide contextual information about affected the neurons, including the neuromuscular junction (Robert Burgess, Jackson Laboratory). In addition, mouse models are the ideal platform for preclinical investigations of therapeutic approaches, including gene therapy to modulate mutant gene expression. An important complement to model system study is direct analysis of ARS function in patient cells. A novel assay involves incubation of patient cell extracts with heavy and light amino acids, followed by quantification of aminoacylation using mass spectrometry (Marisa Mendes, VU University Medical Center).

The dominant nature of ARS-associated CMT alleles means that individuals carrying such alleles in a heterozygous genetic background will express the disease, leading to a relatively high prevalence. ARS diseases associated with recessive genetics can have a high prevalence if they are identified in patients who are compound heterozygotes, or a low prevalence if they are homozygous recessives identified in a closed population where genetically related individuals intermarry. One example of the latter is type 3 Usher syndrome, where a single mutation in the *HARS* gene presents as a progressive sensorineuropathy in which individuals lose hearing and sight in the first and second decades of life [35]. The initial report of this disease featured relatively few patients and focused on the sensorineural defects. However, a more comprehensive clinical study of a larger group of affected children showed that many children experience bouts of acute febrile illness, and many have died from acute respiratory distress (Victoria Siu, London Health Sciences Ontario). Insights into how histidyl-tRNA synthetase (HARS) may be connected to Usher type 3 disease are also emerging from a zebrafish model, where down-regulation of HARS expression during early development leads to specific defects in visual and auditory system development, including in the mechanochemical sensing system (neuroblasts) that is part of the fish lateral line (Ashley Waldron, University of Vermont). Another rare disease where ARS mutations are implicated is pulmonary alveolar proteinosis, which is linked to mutations in the gene encoding the mitochondrial methionyl-tRNA synthetase (MARS2) [36]. While initial characterization suggests that the mutations lead to a loss of function, the work is ongoing (Marc Mirande, Université Paris-Saclay).

Some of the first mutations that were linked to human diseases were identified in mitochondrial ARS genes, and this remains an active area of investigation. Study of these diseases is complicated by the fact that many of the affected patients are compound heterozygotes, inheriting distinct defective alleles from both parents. A priori, one would expect that loss of function in any of a number of mitochondrial ARSs would have a deleterious effect on mitochondrial function, but the related diseases have distinct presentations. One of the earliest ARS-disease correlations linked mutations in the gene encoding mitochondrial aspartyl-tRNA synthetase (*DARS2*) to the disease progressive neurological disease leukoencephalopathy with brainstem and spinal cord involvement and lactate elevation (LBSL). Patients with this disease present with a wide array of neurological symptoms, including spasticity, ataxia, cognitive impairment, and autism. A powerful approach for these diseases involves using Cre-Lox recombination to generate mouse lines with selective knockout in either neurons or astrocytes (Christine Nemeth, Johns Hopkins). Intriguingly, the two lines show different phenotypes, with the line featuring neuronal knockout showing a more drastic disruption of brain morphology than the astrocyte line. Recently, a limited number of cases with compound heterozygous mutations in *MARS2* were reported, and these patients presented with diverse symptoms including developmental delay, poor growth, and sensorineural hearing loss [37]. By use of CRISPR technology, a mouse model of the *MARS2* missense mutation was created. While the mutant mice did exhibit gross motor deficiencies, liver cells from this model did exhibit a decrease in mitochondrial complex 1 protein, signaling mitochondrial dysfunction (Bryn Webb, Icahn School of Medicine). Mutations identified in the *YARS2* gene encoding mitochondrial tyrosyl-tRNA synthetase have also been identified, and these are linked to Leber’s hereditary neuropathy (Min Xin Guan, Zhejiang University). The reduced protein stability and reduced ARS dimer formation of these mutants appear to exacerbate the effect of other mitochondrial genome mutations (i.e., in the *ND4* gene), leading to a reduction in the accumulation of mitochondrial oxidative phosphorylation complexes.

## 7. tRNA Synthetases as Therapeutic Targets

Owing to their universal distribution in all living cells and their fundamental role in interpreting the genetic code, ARSs represent outstanding targets modulating the growth of cells. Consistent with this proposition, a number of natural products (including pseudomonic acid, borrelidin, cladosporin, and others) have been characterized for their anti-ARS activity, particularly as antimicrobial agents [38]. With the exception of the topical antibiotic mupiricin (a derivative of pseudomonic acid), natural product derivatives have not found great application in the clinic, but a very successful program based on boron chemistry led to the development of a class of potent leucyl-tRNA synthetase inhibitors [39]. The most successful of these, Tavaborole (Novartis International AG, Basel, Switzerland), is a topically applied Food and Drug Administration (FDA)-approved treatment for onychomycosis, which is characterized by a fungal infection of the nail and nail bed.

The past success of Tavaborole has encouraged efforts to apply this scaffold to the development of therapeutics against gram negative organisms, but those efforts have not yet been successful. Nevertheless, the long history of antibiotic research programs in major pharmaceutical companies targeting the ARSs served to confirm that these enzymes can be readily targeted by small molecule therapeutics. Building on the well-known and high affinity non-hydrolyzable aminoacyl-sulfamoyl adenosines, one group is developing a class of *N*-leucinyl benzenesulfonamides to target bacterial LeuRS (Michael Charlton, Oxford Drug Design). The compounds have IC50s in the nanomolar range, and some derivatives possess good selectivity for the *E. coli* enzyme over the human. *Mycobacterium tuberculosis* is a high profile antimicrobial target, and by screening chemical libraries through computational chemistry another group has identified derivatives of *N-*benzylidene-*N′-*thiazol-2-yl-hydrazine as lead compounds with modest (~10 μM) IC50s against *Mycobacterium tuberculosis* (Michael Tukalo, Nat. Acad. Sciences, Ukraine). In a third approach, inhibitors to *Pseudomonas aeruginosa* are being identified using the major components of the bacterial translation apparatus as target (James Bullard, University of Texas). In addition to the ribosome and various initiation and elongation factors, a number of tRNA synthetases are being examined as targets. As of this writing initial hits have been reported for methionyl-, histidyl-, and aspartyl-tRNA synthetases [40,41,42].

Therapeutic targeting of the ARSs is not confined to prokaryotic organisms. A major target of ARS therapeutic research that has emerged in recent years is the apicoplast, a non-photosynthetic plastid that is present in *Plasmodia* species and is essential in order for malarial infection to proceed. Of note, some of the ARSs that are encoded by nuclear genes are specifically targeted to the apicoplast [43,44]. Interestingly, inhibitors that target cytoplasmic *Plasmodium* ARSs lead to rapid killing, whereas inhibitors that target apicoplast specific ARSs lead to delayed killing effect (Stuart Ralph, University of Melbourne). Differences between cytoplasmic and chloroplast ARSs in plants can also be readily exploited to develop herbicides. One interesting approach involves the administration of meta-tyrosine (see M. Ibba, above), which is edited to various extents by different PheRSs. In Arabidopsis, the m-Tyr is readily incorporated into the proteomes of mitochondria and chloroplasts, inhibiting their development, but the cytosolic enzyme appears to be more resistant (Liron Klipcan, Hebrew University).

Relative to prokaryotes, therapeutic modulation of eukaryotic ARSs is less well studied, but a number of natural products have been characterized that affect humans ARSs, often in unexpected ways. One of best characterized is halofuginone (HF), derived from the natural quinazolinone alkaloid februgine. Halofuginone is a potent inhibitor of prolyl-tRNA synthetase (EPRS in humans), inhibits collagen synthesis, and is approved for use as a coccidiostat in veterinary medicine [44]. What is arguably most interesting about HF is that relatively low concentrations induce amino acid starvation pathways, leading to reduction of inflammation and suppression of specific pathways in the immune system (Malcolm Whitman, Harvard School of Dental Medicine). The specific TARS inhibitor borrelidin exhibits many of these same effects. Going forward, it will be critical to determine how general these properties are across diverse ARS inhibitors. In at least one exciting new example, splice variant-based fragments of ARS [9] are being investigated for therapeutic potential. In these experiments, constructs based on the HARS N-terminal WHEP domain are being tested for their ability to reverse the tissue damage associated with a bleomycin-induced mouse lung injury model (Leslie Nangle, aTyr Pharma). Initial data suggest that the splice variants promote at least some degree of restoration of tissue homeostasis.

## 8. Aminoacyl-tRNA Synthetase Engineering in Synthetic Biology

The key role that ARSs play in interpreting the genetic code renders them exceptionally important translational components whose modification allows incorporation of non-standard and non-natural amino acids. In the closing keynote lecture that heralded the last day of the meeting, Dieter Söll (Yale University) provided an overview of how special features of the decoding machinery (including the non-standard amino acids, unusual coding assignments, and unusual tRNAs) discovered in the tRNA synthetase field laid the groundwork for elements of the current synthetic biology movement. One can imagine two broad strategies for incorporation of non-natural amino acids into proteins. The first involves development of “orthogonal” aminoacylation systems that use modified tRNA synthetases and tRNAs that interact only minimally with the native aminoacylation systems. Earlier in the meeting, the results of engineering studies on the pyrrolysine system were presented, emphasizing the promise of this system as a platform for expanding the range of non-natural amino acids that can be incorporated (Yuki Yokoyama, U. Tokyo). The development of orthogonal systems will also benefit from the ability of special tRNAs that do not interact with standard host ARS, and a number of these have been identified from both non-standard selenocysteine variants, and from so called “allo tRNAs” with non-standard arm and loop lengths (Takahito Mukai, Yale University).

A rich foundation of work on the editing reactions and the chemistry of aminoacylation has also been important in advancing the frontiers of ARS driven synthetic biology. Many years of research on TyrRS, whose half-of-sites reactivity still attracts research interest (Steven Weeks, KU Leuven), have laid the groundwork for aminoacylation of *D*-amino acids (Eric First, Louisiana State University); major challenges remain in convincing the elongation factors and ribosome to accept these substrates. TyrRS is not a standard editing ARS, but other ARSs that aminoacylate hydrophobic amino acids (including isoleucine, valine, leucine, and threonine) are. Knowledge of the kinetic mechanisms employed by these enzymes to edit out near cognate amino acids is being used to engineer IleRS to accept trifluoroethylglycine as substrate (Ita Gruić-Sovulj, University of Zagreb). Alternatively, one can use in vitro RNA selection technology to dispense with the protein-based aminoacyl-tRNA synthetase altogether. By continuously improving and integrating additional features into an original aminoacylating ribozyme (the “flexizyme” [45]) it is now possible to introduce much of the non-ribosomal derived protein chemistry into function-specific designed peptides, including methylated amino acids, cyclization derivatives, and even variations on the standard peptide bonds (Hiro Suga, University of Tokyo). While these reactions are currently limited to cell free systems, the ability to automate many of the steps increases the throughput of these reactions, speeding the development of new peptide therapeutics.

A current and critical challenge of growing importance is deducing the specific impact of individual protein translational modifications that are introduced by kinases, acetylases, and other enzymes. In a living cell, such modifications may involve only a small fraction of the total pool of a particular protein, making an assessment of their functional impact difficult. By combining the modification of aminoacyl-tRNA synthetases with chemical biology, it becomes possible to introduce post-translationally modified amino acids into target proteins in a site-specific fashion. A key development in this area was the ability to introduce phosphoserine at individual residues [46]. To expand the range of modifications, the phosphoserine is first converted to dehydroalanine. By use of metal conjugation one can attach alkyl halides, thereby creating a new carbon-carbon bond (Hee Sung Park, KAIST). This dramatically increases the range of possible of epigenetic marks that can be introduced. While these developments all represent important milestones towards the goal of wide-scale protein engineering, full implementation demands a deeper understanding of the constraints imposed by recoding activities in the context of a living cell. Current work in this area is focusing on the “context dependence” of recoding and suppression experiments, as it is manifested by DNA sequence context, mRNA secondary structure context, and even the structural context of the resulting protein (Lital Alfonta, Ben-Gurion University; John Fisk, Colorado State University). In spite of these challenges, the revolution in synthetic biology catalyzed by the intelligent engineering of tRNA synthetases is beginning to realize some long sought-after goals. For example, it will soon be possible to produce human kinases in bacterial systems that are multiply phosphorylated—rather than with phosphomimetic residues—and other proteins that are activated by the presence of multiple acetylations (Patrick O’Donogue, U. Western Ontario). 

## 9. Looking Backward, Looking Forward

We have come a long way since the first ARS meeting (Autrans, France, 1990), when the implications of the two distinct classes of ARS enzymes were being debated. Key questions that were raised during after-hours discussions at that meeting were: (1) why two classes of approximately equal members?; (2) Did the two classes emerge at the same time, or did one predate the other?; (3) What is the relationship of the amino acids to the two classes?; (4) How is the genetic code related to the evolution of tRNA identity? A special session at Clearwater showcased some of the theoretical and experimental work that is addressing the evolution of aminoacylation systems. One fruitful line of investigation has been to examine the properties of minimalist class I and class II ARS catalytic domains (“Urzymes” [47]) that were derived from the Ohno-Rodin hypothesis; this posits that both classes were encoded from the complementary strands of an early primordial “master” duplex (Charles Carter, UNC Chapel Hill). Other theoretical work has addressed relationships between the earliest amino acids, RNA acceptor duplexes, and primordial, simplified versions of the genetic code (Hy Hartman, MIT). Any scenario for understanding the evolution of protein synthesis requires a model for the evolution of the ribosome. By use of knowledge gained from rRNA sequence alignments and contemporary structures, it is possible to devise a plausible model of ribosome evolution that starts from a rudimentary peptidyl transferase center and builds outwards (Loren Williams, U. Georgia). Of course, a challenging aspect of any such model is how to account for the simultaneous evolution of a template-directed coding system.

**Figure 2:**
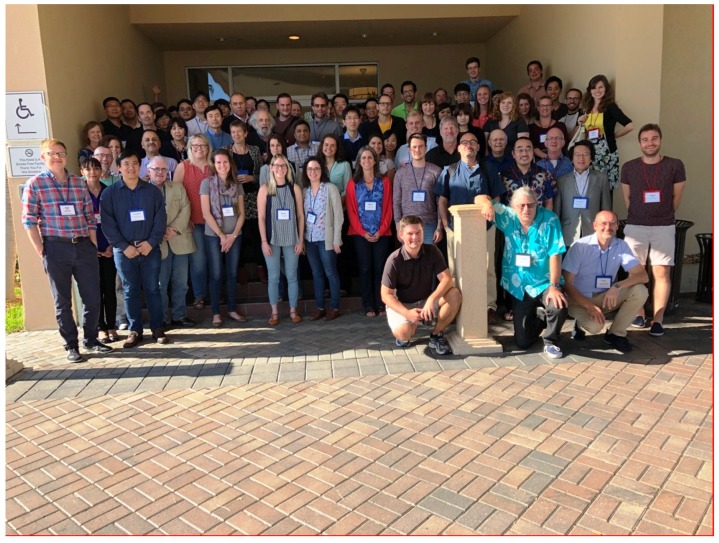
Group photograph of the participants at the Clearwater AARS meeting.

Several main concepts emerging from the meeting provide a view for the significant directions in which the field is heading. First, as seen with other major protein families in the proteome, the special functions of tRNA synthetases emerge from many sources of diversity, including duplications of ARS genes, diverse use of multiply spliced forms, and the vast universe of post-translational modifications that are added and removed in response to physiological conditions. This structural diversity provides a pool of molecules that can be targeted to different compartments in response to physiological stimuli, or even secreted from the cell altogether to take on a largely signaling function. Important clues to how this functional diversity operates on the molecules can be obtained from knowledge of the ARS-interactome, which is emerging from large scale high-throughput mass spectrometry studies. When these interactions become perturbed by inherited mutations in ARS genes, complex human diseases are the result; altered proteostasis is certainly one component of the pathophysiology but is unlikely to be the whole explanation. Rather than merely providing aminoacylated tRNA precursors for protein synthesis, ARSs appear to be tightly integrated into the biological machinery that ensures that protein synthesis is tightly regulated by the availability of nutrients and energy and kept under limits whenever the cell is subjected to infection, redox stress, and other physiological stresses. As work continues on these fascinating enzymes, how these sensing and control functions are manifested will surely be a topic of intense exploration. The Clearwater meeting (Figure 2) set the agenda for this work, but new directions are sure to emerge in the interval between the present and the next gathering. The next aminoacyl-tRNA synthetase meeting will be held in Hangzhou, China, and is tentatively scheduled for November 2019. The principal organizers will be Min-Xin Guan and Jiquing Ling.
